# BDNF Val66Met Polymorphism Reduces the Fatigue-Like Effects of 5-Fluorouracil on Voluntary Wheel-Running Activity in Mice

**DOI:** 10.3389/fnbeh.2022.880969

**Published:** 2022-04-26

**Authors:** Brian S. Wolff, Hannah R. Allen, Li Rebekah Feng, Leorey N. Saligan

**Affiliations:** National Institute of Nursing Research, National Institutes of Health, Bethesda, MD, United States

**Keywords:** BDNF (brain derived neurotrophic factor), Val66Met polymorphism, Val66Met mice, voluntary wheel running activity, chemotherapy, 5-fluorouracil (5Fu)

## Abstract

Fatigue is a persistent and debilitating symptom following cancer treatments such as chemotherapy. Recent clinical studies have suggested a common single-nucleotide polymorphism of brain-derived neurotrophic factor (BDNF), Val66Met (rs6265), may be related to the severity of fatigue following cancer treatment. In this study, we tested transgenic mice homozygous for the human Val66Met BDNF gene and wild-type controls. We injected three doses of 5-fluorouracil (5FU) as a model of chemotherapy treatment, and we used changes in voluntary wheel running activity (VWRA) as a measure of fatigue-like behavior. Prior to 5FU injection, we found that during the baseline wheel-running period, the Val66Met mice lost more weight than WT controls. We next administered 5FU and saw a robust fatigue-like phenotype that lasted about 2 weeks. During the first week, the fatigue-like phenotype was less severe in the Val66Met mice and unrelated to the age of the mice. In contrast, during the second week after 5FU treatment, the fatigue-like phenotype was unrelated to the BDNF genotype but was more severe in middle aged mice compared to young mice. We conclude that the BDNF polymorphism may play a direct, protective role against chemotherapy-induced fatigue.

## Introduction

Cancer treatments can produce debilitating symptoms that have a profound impact on patients’ quality of life. The most common, severe, and consistently reported side effect of chemotherapy is fatigue (O’Regan et al., 2019), which is defined as a sense of tiredness or exhaustion that is not proportional to activity nor sleep. No approved therapies for cancer-related fatigue (CRF) have been developed, in part because the etiology explaining the development of the symptom is poorly understood.

Brain-derived neurotrophic factor (BDNF) is an important signaling molecule in the central nervous system known for its role in neuronal growth and plasticity. The Val66Met single nucleotide polymorphism (SNP) of BDNF involves the substitution of a methionine for a valine residue in the prodomain of BDNF, and it affects the trafficking and release of BDNF (Egan et al., 2003). The prevalence of the met allele varies widely across populations, with estimates ranging from 0.5% in Sub-Saharan Africa to 43.6% in Asia (Petryshen et al., 2010). The polymorphism is most widely known for its association with higher rates of depression and mood disorders (Notaras et al., 2015; Youssef et al., 2018), but recent studies have found a protective effect of the Val66Met polymorphism on cancer-related fatigue. Specifically, one study found a relationship between the Val66Met polymorphism and fatigue in prostate cancer patients after undergoing radiation therapy (Feng et al., 2020) and other studies found a relationship between the Val66Met polymorphism and chemotherapy-related cognitive impairment in patients undergoing chemotherapy treatments for breast cancer (Ng et al., 2015; Tan et al., 2019).

A transgenic mouse model expressing the human Val66Met polymorphism of BDNF was developed and published in 2006 (Chen et al., 2006). Mice with the Val66Met polymorphism show a number of differences from wild-type mice, including increased food intake and increased bodyweight, increased anxiety and depression-like behavior, and a blunted response to antidepressant drugs like fluoxetine (Chen et al., 2006; Bath et al., 2012a,b; Liu et al., 2012; Yu et al., 2012; Ieraci et al., 2020).

Though the Val66Met polymorphism appears related to fatigue symptoms based on clinical observational data, animal models allow for more controlled study of this phenomenon. In this study, we used a mouse model to test whether mice with the Val66Met polymorphism are protected from fatigue-like effects of the commonly used chemotherapy drug, 5-fluororuracil (5FU), and assess whether age and sex played a role in modulating this effect. Similar to our previously published mouse model of radiation-induced fatigue (Wolff et al., 2017), mice in this study received three daily injections of 50 mg/kg 5FU, and we measured changes in voluntary wheel running activity (VWRA) as a fatigue-like outcome measure.

## Materials and Methods

### Ethics

This study was approved by the National Heart Lung and Blood Institute (NHLBI) Animal Care and Use Committee (protocol H-0288) of the National Institutes of Health (NIH), Bethesda, Maryland, United States. All aspects of animal care in this study were compliant with The Guide for the Care and Use of Laboratory Animals (2011).

### Animals

Mice were individually housed in running wheel cages with *ad libitum* access to food and water, on a 12:12 h light cycle, and at roughly 22.2°C and 50% humidity. We used a transgenic BNDF mouse model on a C57BL/6N background, which was first described in detail in 2006 (Chen et al., 2006). Mice included in the study were homozygous for either the human Val66Met SNP of BDNF or for the wild-type mouse BDNF gene (WT). There were 76 mice in total, and we included mice in two age categories: young (54–131 days old at the start of the study) and middle-aged (261–373 days old at the start of the study). Breeder mice were initially provided to us by Dr. Francis Lee’s group at Weill Cornell, and experimental mice were subsequently bred in-house, with littermates included as much as possible. Mice were genotyped by Transnetyx (Cordova, TN, United States) from ear biopsies. Exact numbers of each combination of sex, genotype, and age were determined by their availability and are listed in Table 1.

**TABLE 1:** Group sizes for all experiments.

Sex	Genotype	Age category	Sample size
Female	WT	Middle-aged	*n* = 9
		Young	*n* = 9
	Val66Met	Middle-aged	*n* = 12
		Young	*n* = 7
Male	WT	Middle-aged	*n* = 8
		Young	*n* = 12
	Val66Met	Middle-aged	*n* = 10
		Young	*n* = 9

### Experimental Timeline

All 76 mice were transferred from normal plastic cages into running wheel cages on the first day of the study, and VWRA was recorded throughout the study. Body weights were recorded immediately before entry into the running wheel cages and every 7 days thereafter. After 2 weeks of baseline VWRA, mice received their first set of injections. Starting either 3 or 4 weeks after the first injection, mice then received their second set of injections. Twelve mice were euthanized 2 weeks after initiating the second set of injections; the rest were instead euthanized 6 days after.

For 37 of the 75 mice, the first set of injections were 5FU, and the second set were saline. For the other 39 mice, the first set of injections were saline, and the second set were 5FU. The injection order (whether the mouse received 5FU or saline first) was balanced for each combination of sex, genotype, and age category, or as close as possible for odd numbers. The injection order was included as a factor in the statistical analysis.

### Fatigue Model

Fatigue-like behavior was induced by a set of three intraperitoneal injection of 50 mg/kg 5-fluorouracil (5FU), or with saline injections used as a control. Each set of three injections was one injection per day for three consecutive days. Mice were excluded from the study if they lost more than 20% of their body weight after injection.

### Voluntary Wheel Running Activity

Wheel rotations were recorded in 1-min intervals, 24-h per day, by vertically oriented home cage running wheels (Lafayette Neuroscience, Indiana, United States). VWRA was quantified as “active time,” which is the number of minutes during which the wheel rotated. In previous studies, we have shown that active time is a more stable measure than running distance or running speed (Wolff et al., 2018). Mice were excluded from the study if they did not consistently use the running wheel.

### Open Field Test

Mice were allowed to explore an opaque white arena (45 × 45 cm) under dim light for 20 min. All mice were tested between 1:00 and 4:00 p.m. and animal testing order was designed to balance experimental factors (sex, age, genotype, treatment) across the time period as best as possible. An overhead camera recorded all trials. Distance and center time measurements were calculated from the video using ANY-maze software (Stoelting Co., Wood Dale, IL). Center time was expressed as the percent of the total time during which the center of the mouse was within the 25 × 25 cm center area of the arena. Between testing different animals, arenas were cleaned with ethanol, which were completely dry before starting the next trial.

### Data Analysis

Data were arranged and plotted using *python* 3.9.6. Aggregate statistics were calculated using pandas 1.3.2, *t*-tests were conducted using *scipy* version 1.7.1, and plots were generated using *matplotlib* version 3.4.3 and *seaborn* version 0.11.2. Effect sizes are reported as Cohen’s d. Error bars display standard errors of the mean. For box-and-whisker plots, the boxes report quartiles, and whiskers report the extent of data located within 1.5 times the interquartile range. All *t*-tests are two-sided Welch’s *t*-tests.

Linear regression models were fit using *R* version 3.6.1. When analyzing repeated measures, mixed effects models were fit using *lme4* version 1.1.21, and they included a random intercept for the animal ID. When no repeated measures were being evaluated, linear regression models were fitted using base *R* packages (*stats*). Appropriateness of each model fit was judged using standardized residual plots, q-q plots, and Levene’s test for homogeneity of variance in *car* version 3.0.2. Raw body weight numbers and open field center times were log-transformed to better fit the models. Significance calculations for main effects and interactions were performed using *multcomp* version 1.4.17. All model formulas are included in Supplementary Material.

## Results

### Baseline Measurements

Prior to 5FU treatments, mice spent 2 weeks housed in running wheel cages. We evaluated the average daily VWRA (“baseline VWRA”) and the percent change in body weight (“baseline weight change”) over the 2 weeks, as well as body weight (“baseline body weight”) at the end of the 2 weeks. One mouse was removed from the study due to a sudden drop in VWRA during the baseline period.

#### Baseline Voluntary Wheel Running Activity Was Higher in Younger Mice, but Not Significantly Different Between Genotypes

**FIGURE 1:**
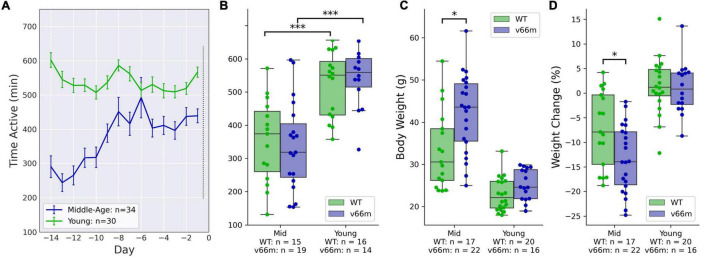
Mice at baseline. Mice ran freely in running wheel cages for 2 weeks before receiving any injections. **(A)** VWRA (time active) on the running wheels for each baseline day. The young mice consistently ran more than middle-aged mice. **(B)** For both Val66Met (“v66m”) and WT, young mice had higher average baseline VWRA than middle-aged (“Mid”) mice (*p* < 10^– 3^), but there were no significant baseline differences between Val66Met and WT mice. **(C)** The Val66Met mice were larger than WT controls. Weight differences between genotypes were statistically significant when comparing only middle-aged mice (*p* = 0.0059). **(D)** Middle-aged but not young mice lost weight during the 2 weeks of baseline wheel running. The middle-aged Val66Met mice lost more weight than their WT controls (*p* = 0.016), even after adjusting for their higher initial bodyweight (*p* = 0.0011). *p < 0.05, ^***^p < 0.0005.

Young mice showed higher VWRA than middle-aged mice throughout the baseline period (Figure 1A). A linear regression model testing for main effects of age, sex, and genotype on VWRA indicated a significant effect of age (*p* < 10^–4^) but not sex (*p* = 0.12) nor genotype (*p* = 0.71). *Post hoc* comparisons showed significant VWRA differences between middle-aged and younger mice with the Val66Met (*d* = 1.35, *t*_32_ = 5.26, *p* < 10^–4^) or WT (*d* = 1.24, *t*_30_ = 4.19, *p* < 10^–3^) genotype (Figure 1B).

#### Bodyweight Was Higher in Val66Met Mice in Both Age Groups

Because the middle-aged mice were so much larger, statistical analyses were done separately for each age group. A linear regression model for baseline bodyweight showed significant main effects of genotype and sex in middle-aged mice (genotype: *p* = 0.034, sex: *p* = 0.0013) and young mice (genotype: *p* = 0.002, sex: *p* < 10^–3^). *Post hoc* comparisons (Figure 1C) showed that in the middle-aged mice, Val66Met mice were significantly heavier than WT (*d* = 0.88, *t*_38_ = 2.93, *p* = 0.0059); in the younger set of mice, Val66Met mice were heavier, but the direct comparison was not statistically significant (*d* = 0.59, *t*_35_ = 1.81, *p* = 0.079).

#### Val66Met Mice Lost More Weight During Voluntary Wheel Running Activity Than Wild-Type Mice

We used a linear regression model to evaluate how baseline weight change was affected by sex, genotype, baseline body weight, and baseline VWRA; the model included interaction terms for sex, genotype, and initial bodyweight. We found very large and significant effects of VWRA (*p* < 10^–3^) and genotype (*p* = 0.0011) on weight change. We tested alternate regression models with more or fewer interaction terms, and they consistently showed significant main effects of genotype and VWRA; some showed significant main effects for sex and initial bodyweight (as well as interaction terms), but they were not consistent across models making it difficult to conclude significance. *Post hoc* comparisons (Figure 1D) showed that for middle-aged mice, the Val66Met mice overall lost significantly more weight than WT controls (*d* = -0.79, *t*_38_ = 2.55, *p* = 0.016).

### Chemotherapy Model

Mice were administered a series of three injections of either saline or 50 mg/kg 5FU over three consecutive days, and VWRA was tracked for up to 3 weeks after. Four mice were removed from the study due to excessive weight loss after 5FU injections.

#### 5FU Induced a Fatigue-Like Reduction in Voluntary Wheel Running Activity

**FIGURE 2:**
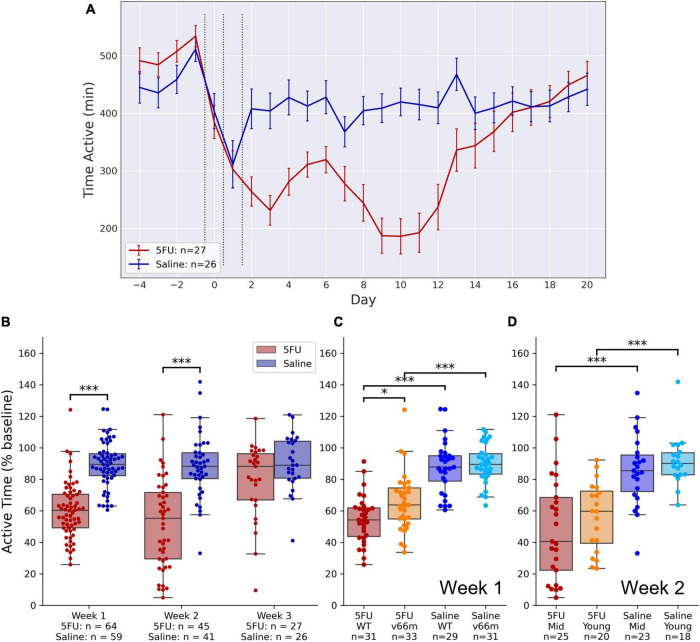
VWRA after 5FU injection. Mice were injected with 50 mg/kg 5FU or saline once per day for 3 days, and VWRA was tracked for 3 weeks. **(A)** VWRA (time active) on the running wheels for each baseline day. The 5FU group shows a clear fatigue-like phenotype with VWRA nadirs during the first week and the second week. Group differences in VWRA appear to end during the third week. **(B)** The fatigue-like phenotype is expressed as the change in VWRA relative to baseline. VWRA differences between the 5FU and saline groups are statistically significant during the first week (*p* < 10^– 16^) and the second week (*p* < 10^– 8^) but not the third week (*p* = 0.062). **(C)** The first week after receiving 5FU injection, the fatigue-like phenotype is less pronounced in Val66Met mice than in WT controls (*p* = 0.012). **(D)** The second week after receiving 5FU injection, the fatigue phenotype is less pronounced in young than middle-aged (“Mid”) mice. *p < 0.05, ^***^p < 0.0005.

As shown in Figure 2A, there was an immediate drop in VWRA that reached a nadir on day 3, then a subsequent drop in VWRA the second week after injection that reached a nadir on days 8–11. A recovery period occurred during the third week, roughly on day 14, For the statistical analysis, VWRA totals were averaged across “week 1” (days 2–6), “week 2” (days 7–13), and “week 3” (days 14–20). The totals were then normalized to the average across the 4 days prior to injections (days –4 through –1). A linear mixed model of VWRA indicated significant main effects for both 5FU dose (*p* = 0.0031) and time (*p* < 10^–9^) as well as their interaction (*p* = 0.0032). *Post hoc* pairwise comparisons (Figure 2B) showed a significant reduction in VWRA for the 5FU group during week 1 (*d* = 1.33, *t*_123_ = 9.91, *p* < 10^–16^) and week 2 (*d* = 1.19, *t*_85_ = 6.89, *p* < 10^–8^). While there was a reduction in week 3 as well, the reduction was not statistically significant (*d* = 0.52, *t*_52_ = 1.91, *p* = 0.062).

5FU also induced weight loss in both young and middle-aged mice (Supplementary Figure 1). The difference in body weight between 5FU and saline groups was largest at week 1 (middle-aged: *d* = 1.38, *t*_68_ = 7.81, *p* < 10^–10^; young: *d* = 1.32, *t*_56_ = 6.45, *p* < 10^–7^), but still significant at week 3 (middle-aged: *d* = 0.75, *t*_44_ = 2.59, *p* = 0.014; young: *d* = 1.03, *t*_31_ = 2.94, *p* = 0.0095).

#### Week 1 Fatigue-Like Behavior Is Related to Genotype but Not Age

During the first week after injections (Week 1), a linear mixed model of VWRA showed significant main effects of 5FU dose (*p* < 10^–4^) and genotype (*p* = 0.0065) with a significant interaction between the two (*p* = 0.022). There were no significant effects of sex (*p* = 0.93), age (*p* = 0.26), or dose order (whether the mouse first received 5FU or saline, *p* = 0.37). *Post hoc* comparisons (Figure 2C) showed significant reductions in VWRA for 5FU-treated vs. saline-treated Val66Met (*d* = 1.24, *t*_63_ = 6.28, *p* < 10^–7^) and WT mice (*d* = 1.46, *t*_59_ = 8.04, *p* < 10^–10^). Importantly, *post hoc* comparisons also showed that Val66Met mice had significantly higher VWRA than WT mice after 5FU treatment (*d* = 0.62, *t*_63_ = 2.60, *p* = 0.012), but not after saline treatment (*d* = 0.14, *t*_59_ = 0.52, *p* = 0.60).

#### Week 2 Fatigue-Like Behavior Is Related to Age but Not Genotype

During the second week after injections (Week 2), a linear mixed model of VWRA showed a significant main effect of injection order (*p* = 0.0037). To simplify the analysis, we excluded data from the second set of injections, and instead used a linear regression model with no random effects using data only from the first set. We found significant effects of 5FU dose (*p* = 0.0012) and age (*p* = 0.029), but no significant effect of genotype (*p* = 0.87) nor any significant interactions. *Post hoc* comparisons showed significant reductions in VWRA as a result of 5FU treatment in middle-aged (*d* = 1.12, *t*_47_ = 4.64, *p* < 10^–4^) and young (*d* = 1.36, *t*_37_ = 5.64, *p* < 10^–5^) mice, but no significant differences comparing ages within the 5FU (*d* = 0.29, *t*_44_ = 0.99, *p* = 0.33) or saline (*d* = 0.29, *t*_40_ = 0.95, *p* = 0.35) groups (Figure 2D).

#### Open Field Behavior Is Related to Sex, but Not 5FU nor Genotype

**FIGURE 3:**
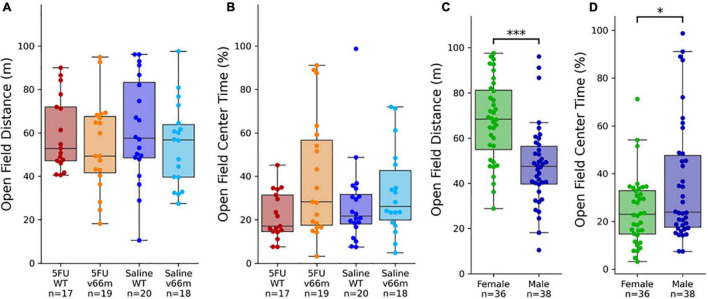
Open field behavior after 5FU injection. **(A,B)** There were no significant effects of 5FU nor BDNF genotype on open field behavior. **(C,D)** Female mice traveled significantly longer distances (*p* < 10^– 4^) and spent less time in the center of the arena (*p* = 0.018) than male mice. *p < 0.05, ^***^p < 0.0005.

A linear regression model found a significant effect of sex on total distance traveled in the open field (*p* < 10^–3^), but there were no significant effects of age (*p* = 0.073), genotype (*p* = 0.27), nor 5FU treatment (*p* = 0.73). A linear regression model found a significant effect of sex on center time in the open field (*p* = 0.020). As with distance (Figure 3A), there were no effects of 5FU treatment nor genotype on center time in the open field (Figure 3B). It is worth noting that the effect of sex on center time was no longer significant if distance traveled were included as a factor in the model, as distance traveled and center time had a very strong negative correlation in our study (Pearson’s *r* = -0.53, *p* < 10^–6^). Pairwise comparisons showed females traveled significantly longer distances than males (Figure 3C, *d* = -0.96, *t*_73_ = 4.67, *p* < 10^–4^) and spent significantly less time than males in the center of the arena (Figure 3D, *d* = 0.54, *t*_73_ = 2.43, *p* = 0.018).

## Discussion

Our main finding is that after treatment with 5FU, the fatigue-like phenotype is less prominent in the Val66Met mice than in the WT controls. This reproduces observations in clinical cancer patients, where the Met allele seems to protect against CRF (Feng et al., 2020). Reproducing this in an animal model is an important step, because correlational findings in a clinical population can be difficult to interpret. Importantly, the animal model advances our understanding of the role of Val66Met in CRF by allowing us to further tease out contributions of variables such as age and sex in a controlled environment, which can be difficult to achieve in clinical studies. The Val66Met polymorphism is associated with other symptoms in humans, including mood disorders like depression (Notaras et al., 2015; Youssef et al., 2018). It is possible that other symptoms may have an impact on self-reported CRF, rather than BDNF being a direct contributor. This is an especially important consideration, as fatigue is considered as part of a network of complex symptom clusters including mood disorders that can be difficult to distinguish in clinical practice (Donovan and Jacobsen, 2007). Further, the prevalence of the Val66Met polymorphism is highly variable across different populations, most notably race, but also subpopulations within racial categories (Petryshen et al., 2010); other differences between these subpopulations, genetic or otherwise, could result in an apparent relationship of the Val66Met SNP with fatigue. The significance of our study is that it provides evidence for a more direct role of the Val66Met SNP on fatigue and fatigue-like behavior. In our mouse model, we compared the behavior of genetically similar mice within an inbred colony, where genetics and other factors like diet, light exposure, exercise, etc. are more controlled. The finding that the Val66Met mice show less fatigue-like behavior suggests that BDNF itself may be mechanistically involved in CRF warrants further study and could point to new targets for intervention.

Interestingly, the 2-week drop in VWRA after 5FU injections appeared to have two phases. The first phase was less severe in mice with the Val66Met polymorphism, but otherwise was consistent between young and middle-aged mice. The second phase was unaffected by the polymorphism but less severe in young mice. One interpretation of this is that the severity of the fatigue-like behavior is affected by the Val66Met polymorphism, but the speed of recovery gets slower with increased age. However, clinical evidence tends to show the opposite, that fatigue is if anything less severe in older patients who receive chemotherapy (Winters-Stone et al., 2008; Bischel et al., 2016). This may be because fatigue is less bothersome in older patients and therefore reported as less severe, but the relationship between age and fatigue needs more study to be properly understood.

Our 5FU-induced fatigue model used three daily doses of 50 mg/kg 5FU in mice on a C57BL/6N background to induce fatigue-like behavior measured by VWRA. Importantly, there appeared to be a full recovery from this dose during the third post-injection week, suggesting this dose did not produce any long-term injury that would affect physical activity. This dose is significantly lower than that used in another study (Mahoney et al., 2013) that used five injections of 60 mg/kg in C57BL/6 mice to produce a very similar outcome. We speculate that fatigue-like outcomes in mice may have a substantial dependence on mouse substrain, much like what we saw in a previous study with a radiation-induced fatigue model, where C57BL/6J mice showed considerably less fatigue-like behavior than C57BL/6N mice (Wolff et al., 2021). Further supporting this is a study by Dougherty et al. (2019) that used the same dosing paradigm as Mahoney et al. (2013) with C57BL/6N mice, but Dougherty et al. (2019) saw a much more severe fatigue-like behavior with a nearly complete cessation of VWRA.

Our baseline VWRA and body weight results match well with what exists in the scientific literature. Earlier studies found that older mice run less than younger mice (Turner et al., 2005), that Val66Met mice are larger than their WT littermates (Chen et al., 2006), and that there are no difference in VWRA between Val66Met mice and WT (Ieraci et al., 2016). We found the same. However, a novel finding in our study is that in the older mice, the Val66Met mice lost more weight than WT controls during VWRA behavior. The reason for the increased weight loss is unclear. VWRA is commonly used experimentally to study the effects of exercise, and it is well-established that exercise induces increased expression of BDNF (Youssef et al., 2018). An earlier study found that Val66Met mice did not show exercise-related benefits in novelty-suppressed feeding and in the forced swim test, and Val66Met mice had lower levels of hippocampal BDNF expression after exercise (Ieraci et al., 2016). In addition, a recent study showed that there are differences in white adipose tissue in Val66Met mice both before and after exercise (Sandrini et al., 2019). These studies demonstrate a clear effect of the BDNF genotype on exercise-related biological changes, so it is possible exercise-induced weight loss in our study is affected in much the same way. However, it is also possible that the increased weight loss is simply due to their increased baseline bodyweight; there is simply more weight to lose. Our linear regression model did not report baseline bodyweight to have a significant effect on weight loss, but that might be due to a non-linear relationship, insensitivity of the model, and/or high variability in the data.

We did not see differences in open field behavior in mice treated with 5FU, which matches prior studies using 5FU (Dougherty et al., 2019) or doxycycline (Zombeck et al., 2013). However, prior studies comparing Val66Met mice to WT have found an anxiety-like phenotype (Chen et al., 2006; Bath et al., 2012a) that we did not see. In fact, the trend we saw was in the opposite direction, where Val66Met mice were spending more time in the center of the arena. We think this discrepancy may be due to a relative lack of stress, as Yu et al. reported effects of Val66Met on open field center time only in stressed mice (Yu et al., 2012). We did not use a stress model in our study; while mice are individually housed and received three injections, both of which are presumably stressful, there is substantial evidence that VWRA alleviates effects of stress on mice (Huang et al., 2017; Pagliusi et al., 2020).

## Conclusion

In conclusion, we found that the fatigue-like decline in VWRA after receiving 5FU injections is smaller in mice with the Val66Met polymorphism of BDNF. This may indicate promising new directions for studying both the mechanisms behind cancer-related fatigue and possible clinical interventions.

## Data Availability Statement

The datasets presented in this study can be found in online repositories. The names of the repository/repositories and accession number(s) can be found below: 10.17605/OSF.IO/GQU4N; https://osf.io/gqu4n/?view_only=f5c01c7bebfb47ba946f5fa1ab7036af.

## Ethics Statement

The animal study was reviewed and approved by the National Heart Lung and Blood Institute (NHLBI) Animal Care and Use Committee.

## Author Contributions

BW: conceptualization, methodology, formal analysis, supervision, and writing—original draft. HA: investigation, methodology, and writing—reviewing and editing. LF: conceptualization, and writing—reviewing and editing. LS: resources, conceptualization, project administration, and writing—reviewing and editing. All authors contributed to the article and approved the submitted version.

## Conflict of Interest

The authors declare that the research was conducted in the absence of any commercial or financial relationships that could be construed as a potential conflict of interest.

## Publisher’s Note

All claims expressed in this article are solely those of the authors and do not necessarily represent those of their affiliated organizations, or those of the publisher, the editors and the reviewers. Any product that may be evaluated in this article, or claim that may be made by its manufacturer, is not guaranteed or endorsed by the publisher.
