# Supplementary material for: BDNF Val66Met Polymorphism Reduces the Fatigue-Like Effects of 5-Fluorouracil on Voluntary Wheel-Running Activity in Mice
Source: Front Behav Neurosci. 2022 Apr 26;16:880969. doi: 10.3389/fnbeh.2022.880969 (PMC9087735; doi:10.3389/fnbeh.2022.880969)
Supplement: Supplementary file 2 [file Image_1.pdf]

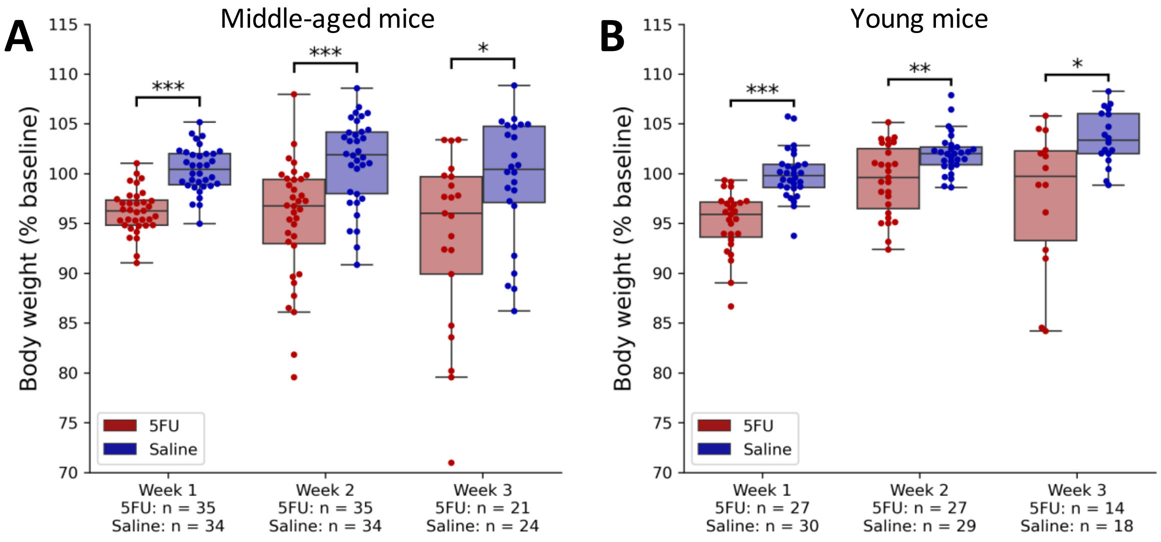


**Supplementary Figure 1**: Weekly bodyweights after 5FU injection. **(A)** Middle-aged 5FU-treated mice had lower body weights than saline controls during the first week ($p < 10^{-10}$), second week ($p < 10^{-4}$), and third week ($p = 0.014$) after injection. **(A)** Young 5FU-treated mice also had lower body weights than saline controls during the first week ($p < 10^{-7}$), second week ($p = 0.0013$), and third week ($p = 0.0095$) after injection.
